# 

**Published:** 1941

**Authors:** 


					f..- 4 I
Vol LVIII. No. 219.
J
. ? ' v A ,
AUTUMN, 1941.
The Bristol
Medico-Chirurgical Journal
F17B1.ISHBD UKDEB THE AUSPICES OV
THE BRISTOL MEDICO-CHIRURGICAL SOCIETY.
Editor: J. A. NIXON, C.M.G., M.D., F.R.C.P.
WITH WHOM ARE ASSOCIATED
E. W. HEY GROVES, D.Sc., M.S., F.R.C.S.
A. E. ILES, O.B.E., M.B., B.S., F.R.C.S.
A. RENDLE SHORT, M.D., B.S., B.Sc., F.R.C.S.
W. MELVILLE CAPPER, M.B., B.S., F.R.O.S.
T. M. LING, M.D., M.R.C.P.
% WATSON-WILLIAMS, M.C., Ch.M., F.R.C.S., Assistant Editor.
BRISTOL:
? W- ARROWSMITH LTD., QUAY STREET & SMALL STREET
Price Three Shillings net.

				

